# Development and validation of entrustable professional activities (EPAs) for rheumatology training in Brazil: the Reuma-EPA project

**DOI:** 10.1186/s42358-026-00565-9

**Published:** 2026-07-17

**Authors:** Claudia Diniz Lopes Marques, Carlos Augusto Ferreira de Andrade, Carolina Müller, Danielle Egypto, Elaine Lira Medeiros, Fabíola Brasil B. Rodrigues, Jozelia Rêgo, Marcelo Pimenta, Maria José Pereira Vilar, Rina Dalva Neubarth, Simone Apenzeller, Sônia Maria A. A. Loduca Lima, Sueli Carneiro, Wilton Ferreira S. Santos, José Eduardo Martinez

**Affiliations:** 1 Universidade Federal de Pernambuco – UFPE, Recife, Brasil; 2 Escola Nacional de Saúde Pública Sergio Arouca/ Fundação Oswaldo Cruz, Rio de Janeiro, Brasil; 3 Universidade Federal do Paraná, Curitiba, Brasil; 4 Universidade Federal da Paraíba, João Pessoa, Brasil; 5 Universidade Federal do Rio Grande do Norte, Natal, Brasil; 6 Centro Universitário do Estado do Pará – CESUPA, Belém, Brasil; 7 Universidade Federal de Goiás - UFG, Goiânia, Brasil; 8 Hospital Geral de Goiânia Dr Alberto Rassi, Goiânia, GO Brasil; 9 Hospital do Servidor Público Estadual de São Paulo -HSPE-SP, São Paulo, Brasil; 10 Universidade Estadual de Campinas, Campinas, Brasil; 11 Universidade Federal do Rio de Janeiro/ Universidade do Estado do Rio de Janeiro, Rio de Janeiro, RJ, Brasil; 12 Escola Superior de Ciências da Saúde, Brasília, Brasil; 13 Faculdade de Ciências Médicas e da Saúde da PUC-SP, São Paulo, Brasil

**Keywords:** Rheumatology, Competency-based medical education, Medical training, Delphi study

## Abstract

**Background:**

Competency-based medical education increasingly guides postgraduate training worldwide. However, rheumatology training in Brazil still lacks a nationally standardized framework to define and assess core professional activities. Entrustable professional activities (EPAs) address this gap by translating competencies into observable clinical practice and supporting supervision, assessment, and progressive autonomy.

**Objective:**

To develop and validate a national EPA framework for rheumatology training in Brazil and to present the conceptual and practical foundations supporting competency-based assessment in the specialty.

**Methods:**

The Reuma-EPA project was conducted by the Education Committee of the Brazilian Society of Rheumatology using a five-phase methodological approach: identification of core professional activities, drafting of preliminary EPAs, internal expert review, internal consistency analysis, and national validation through a two-round Delphi process. Rheumatology educators from accredited residency programs across Brazil evaluated each EPA for relevance, clarity, and suitability for independent execution at the end of training. Consensus was defined as at least 80% of responses rated as 4 or 5 on a five-point Likert scale.

**Results:**

Eleven EPAs were developed and validated, covering the full spectrum of rheumatology practice, including ethical and professional conduct, patient assessment, diagnostic reasoning, laboratory and imaging interpretation, outpatient and inpatient care, use of immunomodulatory therapies, procedural skills, patient safety, and education. Each EPA was mapped to supervision levels and developmental milestones, creating a comprehensive framework that integrates patient-safety considerations and suggested workplace-based assessment tools for Brazilian rheumatology training.

**Conclusion:**

The Reuma-EPA project establishes the first nationally validated EPA framework for rheumatology training in Brazil. By providing an actionable competency-based structure aligned with national regulatory standards, this initiative supports curricular harmonization, transparent assessment, and the progressive development of resident autonomy with patient safety as a central principle. This framework has the potential to strengthen rheumatology training nationally and may serve as a model for other specialties and healthcare systems.

**Supplementary Information:**

The online version contains supplementary material available at https://doi.org/10.1186/s42358-026-00565-9.

## Introduction

Competency-based medical education (CBME) has increasingly reshaped postgraduate medical training by shifting the focus from time-based progression to the demonstration of observable and meaningful professional performance in real clinical settings. Within this paradigm, entrustable professional activities (EPAs), first introduced by ten Cate in 2005 [1], provide a practical framework to operationalize competencies by translating them into units of professional practice that can be entrusted to trainees once sufficient competence is demonstrated. EPAs thus bridge the gap between abstract competency frameworks and day-to-day clinical work, enabling more authentic assessment and progressive autonomy in residency training.

Entrustment decisions are complex and multidimensional, integrating technical competence, clinical reasoning, consistency of performance, professionalism, communication skills, and the ability to recognize personal limits and seek supervision appropriately. These decisions are not isolated from judgments, but longitudinal processes are informed by repeated observations across clinical contexts and supervisors. As such, EPAs function not only as assessment tools but also as educational instruments that promote structured feedback, reflective practice, patient safety, and professional identity formation [2].

Over the past two decades, EPA-based frameworks have been adopted across multiple medical specialties, including internal medicine [3], pediatrics [4], surgery [5], anesthesiology [6], emergency medicine [7], obstetrics and gynecology [8] and psychiatry [9]. These initiatives have defined both generic clinical activities, such as handover of care and clinical reasoning in acute care, and discipline-specific activities that reflect the daily practice of each specialty. However, the development and implementation of EPAs in rheumatology remain relatively recent and heterogeneous. Existing initiatives are often limited to specific institutions, countries, or residency programs, and many training systems still rely predominantly on traditional knowledge-based evaluations rather than structured workplace-based entrustment decisions [10, 11].

In Brazil, the progressive incorporation of competency-based principles into national residency standards has highlighted the need for clearer operational tools to support supervision, assessment, and progressive autonomy in rheumatology training. Despite this need, no nationally validated EPA framework for rheumatology residency training was previously available. To address this gap, the Teaching and Education Committee of the Brazilian Society of Rheumatology initiated the Reuma-EPA project, a national effort to systematically define and validate EPAs tailored to rheumatology practice in the Brazilian context.

Grounded in international CBME principles and aligned with the competency matrix of the National Medical Residency Commission, the Reuma-EPA project establishes a structured, assessment-oriented framework that articulates essential professional activities, supervision levels, developmental milestones, and patient-safety considerations relevant to rheumatology residency training. The objective of this study was to develop and validate a national set of EPAs reflecting the core professional activities of rheumatologists in Brazil, using a consensus-based methodology. By establishing a shared framework for entrustment decisions, this project aims to support curricular alignment, enhance assessment transparency, and promote safe, progressive autonomy in rheumatology training. 

## Methods

### Study design

This methodological study was conducted between 2024 and 2025 by the Education and Teaching Committee of the SBR. The project was designed to develop and validate a national framework of EPAs for rheumatology residency training, ensuring alignment with CBME principles and national regulatory standards. The study followed a structured, multi-phase approach to ensure methodological rigor, transparency, and national applicability.

### Conceptual framework

The Reuma-EPA project was guided by international literature on EPAs [1, 12, 13] and by the revised Miller’s Pyramid [14], emphasizing the integration of knowledge, skills, and professional attitudes into observable and assessable clinical performance. Each EPA was conceptualized as a distinct unit of professional practice so that a rheumatology resident should be able to perform independently and safely by the end of training.

### EPA structure

Each EPA followed a standardized structure to ensure consistency, clarity, and usability across training programs. The components included:

- Title and brief description
- Justification and clinical relevance
- Related Accreditation Council for Graduate Medical Education (ACGME)-based competency domains (medical knowledge, patient care, professionalism, communication, systems-based practice, and lifelong learning
- Competencies addressed: aligned with the competencies defined in the official NMRC competency framework for medical residency in rheumatology [15]. It integrates core knowledge, clinical and procedural skills, professional attitudes, and systems-based practice requirements expected throughout residency training, supporting progressive development of autonomy and safe, high-quality patient care.
- Required knowledge, skills, and attitudes
- Clinical contexts of application and associated risk level
- Expected level of supervision (from 1 - observation only to 5 - ability to supervise others)
- Developmental milestones
- Suggested assessment tools
- Explicit criteria for entrustment and safe autonomy

### Development phases

The methodology followed five structured phases, adapted from CBME models:

1. **Identification of core professional activities**: To inform the identification and prioritization of core professional activities relevant to rheumatology practice, a national survey was conducted among members of the Brazilian Society of Rheumatology (SBR). The objective was to capture practicing rheumatologists’ perspectives on the relative importance of clinical, diagnostic, procedural, professional, and systems-based activities, thereby supporting the content validity of the EPA development process. The survey was developed by the SBR Education Committee and administered electronically using the REDCap platform. It included demographic and professional characteristics and a structured list of professional activities commonly performed in rheumatology practice. Respondents rated the importance of each activity for rheumatology training using a five-point Likert scale (1 = low relevance; 5 = essential). The survey was distributed electronically to SBR members, and participation was voluntary and anonymous. Activities consistently rated as highly relevant or essential informed the selection and initial drafting of the EPAs. Detailed survey items and results are provided in Supplementary Appendix 1.
2. **Drafting of preliminary EPA**: identified activities were systematically translated into preliminary EPAs statements, ensuring that each represented a discrete, observable, and meaningful unit of professional practice. An initial set of ten EPAs was drafted, each structured according to the standardized EPA template described above, including a clear title, scope of practice, associated competencies, expected supervision levels, developmental milestones, and suggested assessment strategies.
3. **Internal review and refinement**: The preliminary EPAs underwent internal review by members of the SBR Education Committee to assess relevance, clarity, scope, redundancy, and alignment with national standards. EPAs with overlapping content or insufficient linkage to observable clinical practice were refined, merged, or reformulated.
4. **Assessment of internal consistency**: Prior to external validation, a structured qualitative analysis was performed to ensure internal consistency. Committee members independently reviewed each EPA using predefined criteria that addressed observability, relevance to rheumatology practice, alignment between competencies and expected supervision levels, clarity of wording, feasibility of assessment in real clinical settings, and consistency between the level of clinical risk and the degree of expected trainee autonomy. Discrepancies were discussed in consensus meetings, resulting in further refinement and reduction of the initial set to ten EPAs. Each member of the Education Committee independently analyzed all draft EPAs using a structured checklist comprising the following guiding questions:

- *Does the EPA describe a real*,* observable unit of professional practice that can be assessed in the workplace?*
- *Is the EPA relevant to the typical scope of rheumatology practice and to resident training objectives?*
- *Are the knowledge*,* skills*,* and attitudes associated with this EPA coherent and proportional to the proposed level of autonomy?*
- *Is the EPA wording clear, concise, and free of ambiguity or overlapping with other EPAs?*
- *Are the supervision levels (1–5) consistent with patient-safety risk and resident progression?*
- *Is there internal consistency between the competencies, milestones, and assessment tools listed?*
- *Does the EPA allow reliable observation and documentation through feasible assessment instruments (e.g., Mini-CEX, DOPS, portfolio)?*

After the individual analysis, the committee met for a consensus discussion to review discrepancies and refine the EPAs. Activities that presented conceptual overlap or weak linkage with observable practice were reformulated or merged. The review led to improvements in clarity, balance among cognitive, technical, and attitudinal domains, and coherence between risk level, supervision, and expected autonomy. This step provided evidence of internal consistency and content validity, ensuring that each EPA represented a meaningful, assessable, and contextually appropriate element of rheumatology training.

5. **Delphi validation**: The refined EPAs were validated through a two-round Delphi process, consisting of one in-person round followed by a subsequent online round. Each EPA was evaluated individually by the Delphi panel using a five-point Likert scale. Panelists were instructed to consider three criteria when rating each EPA: relevance to rheumatology residency training, clarity of wording, and adequacy for independent execution by the end of residency. These criteria guided the overall judgment of each EPA, but were not scored as separate numerical subdomains in the Delphi instrument. Consensus was defined a priori as at least 80% of responses rated as 4 or 5 on the Likert scale. After the first round, quantitative ratings were analyzed together with free-text comments. Items with lower levels of agreement, partial agreement, or substantive qualitative suggestions were reviewed by the coordinating group. Proposed modifications were incorporated when they improved clarity, scope, feasibility, or alignment with the expected competencies of rheumatology residency training. The revised EPA statements were then submitted to the panel in the second Delphi round to confirm consensus and finalize the framework.

### Participants

The Delphi panel consisted of thirty-one members of the Brazilian Society of Rheumatology who were actively involved in rheumatology residency training programs as preceptors, supervisors, or faculty members. Participants had substantial experience in rheumatology education and represented four Brazilian macro-regions, including the North, Northeast, Southeast, and South regions, and included professionals from Amazonas, Paraíba, Pernambuco, Alagoas, Ceará, São Paulo, Rio de Janeiro, Minas Gerais, Paraná, and Rio Grande do Sul. To preserve the independence of the validation process, Delphi participants had not taken part in the previous phases of the Reuma-EPA project, including the initial drafting, preliminary discussions, or internal refinement of the EPA framework. The broader group acknowledged in the manuscript included collaborators who contributed to earlier stages of the initiative, institutional support, dissemination, or complementary educational activities, but they were not part of the Delphi consensus panel. Information on the size of each affiliated training program was not systematically collected and is acknowledged as a limitation.

## Results

### Identification of core professional activities

A total of 120 rheumatologists responded to the national survey distributed to members of the SBR. Respondents represented different regions of the country and a range of professional profiles, including clinicians involved in outpatient care, inpatient care, emergency services, and residency training. The survey aimed to identify and prioritize professional activities considered most relevant to routine rheumatology practice and training.

Overall, activities related to direct patient care, clinical reasoning, and professional conduct were consistently rated as highly important or essential for rheumatology training. The highest importance ratings were observed for structured rheumatologic history taking, accurate musculoskeletal physical examination, ethical and professional conduct, pharmacological management of rheumatic diseases—particularly immunomodulatory and immunosuppressive therapies—and longitudinal outpatient management of patients with chronic rheumatic conditions. Patient and family education, as well as patient safety and quality-of-care practices, were also among the most highly prioritized activities.

Diagnostic reasoning and the appropriate use of complementary investigations were considered central to rheumatology practice. Respondents rated the indication and interpretation of laboratory tests and imaging studies as highly relevant, with greater emphasis on clinical integration of results than on advanced image acquisition. Interpretation of plain radiographs and bone densitometry was consistently rated as more essential than interpretation of advanced imaging modalities, such as computed tomography, magnetic resonance imaging, or scintigraphy, which showed greater variability in importance ratings.

Procedural skills, including arthrocentesis and joint injections, were considered important components of rheumatology training, whereas more specialized procedures, such as musculoskeletal ultrasonography and capillaroscopy, demonstrated wider variability in perceived relevance. Activities related to inpatient care, interprofessional collaboration, consultative practice, and coordination of care across healthcare settings were also rated as important, particularly by respondents involved in hospital-based practice.

### Validation of entrustable professional activities

Following the identification, drafting, and internal refinement phases, an initial set of ten EPAs was submitted to national validation through a structured Delphi process involving educators from SBR-accredited residency programs.

In the in-person Delphi exercise, agreement was high across most evaluated domains. For most EPAs, agreement ranged from 85.7% to 100%. The lowest agreement was observed for “assessment and supervision levels” in EPA 4, which reached 77% agreement and was therefore revised for reassessment. Other domains with comparatively lower agreement were also mainly related to assessment and supervision levels, including EPA 2 (87%), EPA 5 (85.7%), and EPA 9 (89%) (Table 1).

During this process, participants also identified an important gap in the preliminary framework: the absence of an EPA explicitly addressing non-pharmacological treatment strategies in rheumatology. As a result, a new EPA, “Indicate and prescribe non-pharmacological treatment strategies in rheumatology,” was developed and incorporated into the validation process. Revised EPAs and newly incorporated components were subsequently evaluated in a final remote Delphi exercise.

In the final Delphi exercise, EPA 4 reached 100% agreement across all reassessed domains. The newly incorporated EPA on non-pharmacological treatment strategies and EPA 11, which also underwent additional refinement, reached 100% agreement across all evaluated domains. After completion of the Delphi process, all eleven EPAs met the predefined consensus criteria and were retained in the final framework.

Final agreement should be interpreted as consensus after iterative review and refinement, rather than as absence of disagreement during earlier stages of the Delphi process. Agreement was defined as the proportion of participants who answered either “strongly agree” or “partially agree.” Domain-specific agreement across Delphi exercises and final Reuma_EPA framework is summarized in Table 1. Detailed, domain-specific agreement data for each EPA and each Delphi round are presented in Supplementary Appendix 2.

**Table 1:** Final reuma-EPA framework and summary of agreement across Delphi exercises

EPA	EPA title	In-person Delphi agreement range, %	Lowest-agreement domain in the in-person Delphi	Action after in-person Delphi	Final Delphi result
1	Demonstrate ethical, professional, and compassionate patient care, and collaborate effectively within interprofessional teams	93–100	Assessment and supervision levels; associated risks	No final reassessment required	Not applicable
2	Obtain a structured, comprehensive rheumatologic history	87–100	Assessment and supervision levels	Minor refinement based on panel feedback; no final reassessment required	Not applicable
3	Perform and accurately interpret a systematic rheumatologic physical examination	97–100	Assessment and supervision levels	No final reassessment required	Not applicable
4	Manage outpatient care for patients with rheumatic diseases	77–100	Assessment and supervision levels	Revised and submitted to final Delphi reassessment	100% agreement across all reassessed domains
5	Provide inpatient care for patients with rheumatic diseases, including ward care, emergency care, intensive care unit management, and consultative services	85.7–100	Assessment and supervision levels	Minor refinement based on panel feedback; no final reassessment required	Not applicable
6	Appropriately order and interpret diagnostic tests to support diagnosis and longitudinal monitoring of rheumatic diseases	97–100	Assessment and supervision levels; associated risks	No final reassessment required	Not applicable
7	Appropriately order and interpret imaging studies to confirm or refine diagnostic hypotheses in rheumatic diseases	93–100	Developmental milestones	Minor refinement based on panel feedback; no final reassessment required	Not applicable
8	Indicate and prescribe non-pharmacological treatment strategies in rheumatology	Not applicable	Not applicable	Newly developed after identification of a gap regarding non-pharmacological treatment strategies	100% agreement across all evaluated domains
9	Indicate, prescribe, and monitor pharmacological treatment in rheumatology	89–100	Supervision levels	Minor refinement based on panel feedback; no final reassessment required	Not applicable
10	Perform arthrocentesis and joint and periarticular injections	95–100	Associated risks	No final reassessment required	Not applicable
11	Promote patient safety and quality of care, and provide patient and family education on rheumatic disease and its treatment	95–100	Associated risks	No final reassessment required	Not applicable

Note: Agreement was defined as the proportion of participants who answered either “strongly agree” or “partially agree.” Final agreement should be interpreted as consensus after iterative review and refinement, rather than as absence of disagreement during earlier stages of the Delphi process. “Not applicable” indicates that the EPA was not assessed or reassessed in that specific Delphi exercise or that final reassessment was not required

## Discussion

The Reuma-EPA project represents a significant step toward the operationalization of competency-based medical education in rheumatology training in Brazil. By defining and validating a national set of EPAs, this initiative translates abstract competency frameworks into observable and assessable units of professional practice, directly linked to supervision levels, patient-safety considerations, and progressive autonomy. In doing so, it contributes to greater transparency, consistency, and accountability in residency training, aligning educational processes more closely with real-world clinical practice.

The methodological rigor of the project strengthens the credibility and applicability of the proposed framework. The use of sequential development phases, internal consistency analysis, and national Delphi validation ensured conceptual clarity, content validity, and alignment with contemporary educational standards. The deliberate integration of cognitive, technical, and attitudinal domains within each EPA reflects a holistic understanding of professional competence. Furthermore, alignment with the competency matrix of the NMRC reinforces regulatory relevance and supports the potential adoption of the framework across accredited training programs.

The development of EPAs in rheumatology aligns with a broader national and international movement toward CBME, which has progressively transformed postgraduate training paradigms over the past two decades [12, 16]. While the conceptual framework of EPAs is now well established, the operationalization of these activities in specific specialties, particularly in smaller fields such as rheumatology, remains limited and heterogeneous.

Early initiatives in internal medicine played a foundational role in defining methodological standards for EPA construction. Hauer et al. [17] proposed one of the first validated sets of EPAs, demonstrating how entrustment decisions could bridge the gap between theoretical competencies and workplace performance. Rinaldi et al. [18] expanded this approach to a non-Western context, confirming that EPA validation through structured Delphi consensus is feasible and culturally adaptable. Similarly, Valding et al. [3] revealed considerable variability among existing EPA frameworks, reinforcing the need for contextualization to local educational structures and healthcare systems. These findings collectively suggest that a “one-size-fits-all” approach is inadequate — instead, successful EPAs implementation depends on tailored curricular integration and explicit connection with assessment practices.

Evidence from surgical disciplines further illustrates both the potential and the challenges of EPA-based systems. Although EPAs have enhanced the evaluation of procedural competence and autonomy, barriers related to faculty engagement, feasibility, and institutional support persist [19]. These challenges closely mirror those encountered in rheumatology, where clinical workload and educational culture may limit systematic observation and feedback. Nevertheless, surgical literature provides important evidence of the reliability of EPA-based assessments, including strong inter-rater agreement and improved learner accountability.

Within rheumatology, the evidence base remains recent but promising. Alharbi et al. [11] conducted the first comprehensive Delphi validation of EPAs for rheumatology residency training, underscoring the importance of aligning entrustment levels with milestones and patient safety. Their model emphasizes the value of national standardization while preserving flexibility for local adaptation. Chakr et al. [20] documented the first Latin American experience applying EPAs in residency programs, demonstrating that their use enhances residents’ autonomy and fosters a more reflective feedback culture among preceptors. However, both studies highlight persistent challenges, including limited faculty training and the need for longitudinal follow-up to ensure that EPA-based assessment translates into sustained competence.

Taken together, the literature supports the view that EPAs function not merely as assessment tools, but as catalysts for cultural change in postgraduate medical education. They reconceptualize supervision as a dynamic process of progressive trust, grounded in shared understanding between supervisors and trainees. At the same time, a recurring tension emerges between standardization and flexibility: while structured frameworks promote comparability and quality assurance, excessive rigidity may compromise contextual relevance and faculty engagement [12].

A major strength of this study lies in its alignment with international experiences while remaining responsive to the Brazilian healthcare and educational context. The Reuma-EPA project is consistent with global trends in competency-based medical education, particularly in its emphasis on entrustment as a longitudinal, performance-based decision grounded in multiple observations and professional judgment. By clearly articulating expected supervision levels and developmental milestones, the framework fosters a shared mental model between supervisors and residents, which is essential for both patient safety and effective learning.

Building on international experience, the Reuma-EPA Project offers a context-sensitive and nationally endorsed framework that addresses a significant gap in rheumatology education in Brazil. Beyond defining competencies, it provides an actionable pathway for implementing CBME principles in everyday clinical settings. This reinforces the notion that the effectiveness of EPAs initiatives depends less on the number of activities defined and more on their meaningful integration into daily supervision, reflection, and feedback practices.

Several limitations should be acknowledged. First, the Reuma-EPA framework was developed and validated through expert consensus, providing evidence of content validity but not direct evidence of performance in real training environments. The Delphi process allowed structured refinement of the EPA set by experienced rheumatology educators; however, it did not include direct observation of residents, assessment of entrustment decisions in clinical practice, or measurement of learner or patient-related outcomes. Therefore, the present findings should not be interpreted as evidence of feasibility, reliability, implementation effectiveness, or educational impact. Second, the framework has not yet undergone pilot implementation across residency programs. As a result, important implementation outcomes, including acceptability among residents and supervisors, usability in workplace-based assessment, inter-rater consistency, faculty workload, and integration into existing curricula, remain to be evaluated. Future multicenter pilot studies will be necessary to determine how the EPAs function in daily clinical training and whether they support progressive autonomy and more consistent assessment practices. Third, heterogeneity among Brazilian rheumatology training programs may influence implementation. Differences in institutional resources, faculty availability, clinical exposure, patient volume, and prior experience with competency-based medical education may affect how the framework is adopted locally. Programs without permanent rheumatology faculty or with limited supervisory capacity may require external mentorship, regional collaboration, faculty development, and contextual adaptation to ensure safe and meaningful use of the EPAs. Finally, although agreement across Delphi exercises was descriptively assessed, formal statistical testing of stability between rounds was not performed. In addition, relevance, clarity, and adequacy for independent execution were used as guiding criteria for the overall evaluation of each EPA, rather than as separate numerical subscales. These methodological choices should be considered when interpreting the results. Overall, this study establishes an expert-based, nationally developed content framework for rheumatology residency training, but further research is required to generate validity evidence related to feasibility, reliability, implementation, and educational outcomes.

## Conclusion and future directions

The Reuma-EPA project developed by the SBR represents a pioneering national initiative to align rheumatology residency training with international standards of competency-based medical education. Through the systematic identification, validation, and operationalization of rheumatology-specific EPAs this framework translates competency expectations into observable clinical practice, supporting consistent entrustment decisions, structured supervision, and patient-centered care.

By establishing a nationally agreed-upon set of EPAs, the Reuma-EPA Project provides a robust foundation for curricular alignment, workplace-based assessment, and the progressive development of resident autonomy while maintaining patient safety as its central organizing principle. Beyond harmonizing training across programs, the framework promotes a culture of trust, meaningful feedback, and professional accountability, with patient safety as its central organizing principle. As implementation and evaluation progress, this initiative has the potential to strengthen rheumatology training in Brazil and to serve as a scalable model for other specialties and healthcare systems seeking to operationalize competency-based education in a real-world clinical setting.

The next phase of the Reuma-EPA project will focus on implementation and evaluation. Planned steps include pilot testing of the EPAs in selected SBR-accredited residency programs to assess feasibility, clarity, and integration into daily clinical supervision. These pilots will allow refinement of EPAs descriptions, supervision expectations, and assessment strategies based on real-world use.

Faculty development will be a critical component of successful implementation. Training preceptors to observe performance, provide structured feedback, and make defensible entrustment decisions is essential to ensure consistency and educational value. Workshops, online modules, and standardized guidance documents are planned to support faculty engagement and calibration.

Integration with national accreditation and assessment systems represents another important future direction. The Reuma-EPA framework has the potential to inform formative assessments throughout residency and, selectively, summative decisions related to progression or completion of training. Its alignment with the NMRC competency matrix facilitates dialogue with regulatory bodies regarding its use as a complementary assessment strategy.

Although developed for rheumatology residency training, the Reuma-EPA framework is not limited to programs with a formally affiliated rheumatologist. As a published educational framework, it may be used by training programs as a reference to define expected professional activities, identify curricular gaps, structure clinical rotations, and guide workplace-based assessment in rheumatology-related settings. Nevertheless, EPA-based entrustment decisions require qualified supervision and should ideally involve rheumatologists or supervisors with appropriate expertise, either locally or through external collaboration, regional networks, or faculty development initiatives.

Finally, future research should evaluate the educational and clinical impact of EPA-based training in rheumatology, including effects on resident development, supervision practices, and patient safety. Longitudinal studies and mixed-methods approaches will be particularly valuable in understanding how EPAs function as instruments of cultural change within postgraduate medical education.

## Supplementary Information

Below is the link to the electronic supplementary material.

Supplementary Material 1

Supplementary Material 2

## Data Availability

No datasets were generated or analysed during the current study.

## References

[1] ten Cate O. Entrustability of professional activities and competency-based training. Med Educ. 2005;39(12):1176–7. 10.1111/j.1365-2929.2005.02341.x. 16313574

[2] Ten Cate O, Hart D, Ankel F, Busari J, Englander R, Glasgow N, et al. Entrustment Decision Making in Clinical Training. Acad Med. 2016;91(2):191–8. 10.1097/ACM.0000000000001044. 26630606

[3] Valding B, Monti M, Junod Perron N, Frick S, Jaques C, Nendaz M, Gachoud D. Entrustable professional activities for residency in general internal medicine: a systematic review. Swiss Med Wkly. 2022;152:40002. 10.57187/smw.2022.40002. 36473152

[4] Kerth JL, van Treel L, Bosse HM. The Use of Entrustable Professional Activities in Pediatric Postgraduate Medical Education: A Systematic Review. Acad Pediatr. 2022;22(1):21–8. 10.1016/j.acap.2021.07.007. 34256178

[5] Montgomery KB, Mellinger JD, Lindeman B. Entrustable Professional Activities in Surgery: A Review. JAMA Surg. 2024;159(5):571–7. 10.1001/jamasurg.2023.8107. PMC11260519 38477902

[6] Tanaka P, Marty A, Park YS, Kakazu C, Udani A, Pardo M, et al. Defining entrustable professional activities for first year anesthesiology residents: A Delphi study. J Clin Anesth. 2023;88:111116. 10.1016/j.jclinane.2023.111116. 37278050

[7] Passoni Lopes LC, Ferrazini RVS, Costa K, de Albuquerque WL, Carvalho C, Kwan J, et al. Entrustable professional activities, emergency medicine and medical education: a systematic review. Int J Emerg Med. 2024;17(1):112. 10.1186/s12245-024-00699-x. PMC11375971 39237864

[8] Novellino AMMCI. Creation of Entrustable Professional Activities (EPAs) in obstetrics and gynecology for medical undergraduate students. Rev Bras Educ Med. 2021;45(04):9. 10.1590/1981-5271v45.4-20200472.ING.

[9] Pinilla S, Lenouvel E, Strik W, Kloppel S, Nissen C, Huwendiek S. Entrustable Professional Activities in Psychiatry: A Systematic Review. Acad Psychiatry. 2020;44(1):37–45. 10.1007/s40596-019-01142-7. 31732885

[10] Canada TRCoPaSo. Entrustable professional activities for adult rheumatology. 2019.

[11] Alharbi LA, Cheikh M, Alotaibi ME, Alkhotani AA, Alim HM, Almalki F, et al. Developing and Validating Entrustable Professional Activities (EPAs) for Rheumatology Fellowship Training Programs in Saudi Arabia: A Delphi Study. Adv Med Educ Pract. 2024;15:845–56. 10.2147/AMEP.S481977. PMC11416783 39308482

[12] Ten Cate O, Balmer DF, Caretta-Weyer H, Hatala R, Hennus MP, West DC. Entrustable Professional Activities and Entrustment Decision Making: A Development and Research Agenda for the Next Decade. Acad Med. 2021;96(7S):S96–104. 10.1097/ACM.0000000000004106. 34183610

[13] Ten Cate O, Carraccio C, Damodaran A, Gofton W, Hamstra SJ, Hart DE, et al. Entrustment Decision Making: Extending Miller’s Pyramid. Acad Med. 2021;96(2):199–204. 10.1097/ACM.0000000000003800. 33060399

[14] Cruess RL, Cruess SR, Steinert Y. Amending Miller’s Pyramid to Include Professional Identity Formation. Acad Med. 2016;91(2):180–5. 10.1097/ACM.0000000000000913. 26332429

[15] Brasil. Resolução Nº 2, de 7 de dezembro de 2020. Aprova a Matriz de Competências dos Programas de Residência Médica de Reumatologia. In: Médica MdEeCSdESCNdR, editor. Diário Oficial da Uniao [Internet], Nº 235; 09 de dezembro de 2020. Brasília 2020.

[16] El-Haddad C, Damodaran A, McNeil HP, Hu W. The ABCs of entrustable professional activities: an overview of ‘entrustable professional activities’ in medical education. Intern Med J. 2016;46(9):1006–10. 10.1111/imj.12914. 26388198

[17] Hauer KE, Kohlwes J, Cornett P, Hollander H, Ten Cate O, Ranji SR, et al. Identifying entrustable professional activities in internal medicine training. J Grad Med Educ. 2013;5(1):54–9. 10.4300/JGME-D-12-00060.1. PMC3613318 24404227

[18] Rinaldi I, Findyartini A, Widaty S, Hasan I. Identification and Validation of Entrustable Professional Activities in Indonesian Internal Medicine Residency Programs. Acta Med Indones. 2022;54(1):79–96. 35398829

[19] Kitto S, Fantaye AW, Zevin B, Fowler A, Sachdeva AK, Raiche I. A Scoping Review of the Literature on Entrustable Professional Activities in Surgery Residency Programs. J Surg Educ. 2024;81(6):823–40. 10.1016/j.jsurg.2024.02.011. 38679495

[20] Chakr RMSKC, Brenol CV, Xavier RM, Monticielo OA. Insights from a 3-year experience: depicting the flourishing of rheumatologists through entrustable professional activities. Rev Bras Educ Med. 2025;49(1). 10.1590/1981-5271v49.1-2024-0074.ING.

