# Supplementary material for: Development and validation of entrustable professional activities (EPAs) for rheumatology training in Brazil: the Reuma-EPA project
Source: Adv Rheumatol. 2026 Jul 17;66:57. doi: 10.1186/s42358-026-00565-9 (PMC13660143; doi:10.1186/s42358-026-00565-9)
Supplement: Supplementary file 1 [file 42358_2026_565_MOESM1_ESM.pdf]

For the best experience, open this PDF portfolio in Acrobat X or Adobe Reader X, or later.

Get Adobe Reader Now!
