# Supplementary material for: Development and validation of entrustable professional activities (EPAs) for rheumatology training in Brazil: the Reuma-EPA project
Source: Adv Rheumatol. 2026 Jul 17;66:57. doi: 10.1186/s42358-026-00565-9 (PMC13660143; doi:10.1186/s42358-026-00565-9)
Supplement: Supplementary file 2 [file 42358_2026_565_MOESM2_ESM.pdf]

<table>
<tr><th>EPA Number</th><th>1</th></tr>
<tr><th>EPA Title</th><th>Demonstrate ethical, professional, and compassionate patient care, and collaborate effectively within interprofessional teams</th></tr>
<tr><td>Brief description</td><td>The resident demonstrates ethical, empathetic, and professional conduct in rheumatology patient care, promotes person-centered care, respects sociocultural values, and collaborates effectively within interprofessional healthcare teams.</td></tr>
<tr><td>Rationale</td><td>Ethical and professional training is essential to ensure safe, humane, and high-quality care. In rheumatology, compassionate and collaborative practice is critical due to disease chronicity and complexity, requiring empathy, effective communication, and teamwork.</td></tr>
<tr><td>Competency domains</td><td>Medical knowledge
Interpersonal and communication skills
Professionalism</td></tr>
<tr><td>Competencies addressed (based on the Competency Matrix of the National Medical Residency Commission)</td><td><u>First Year</u>
Integrate ethical, moral, social, and economic principles into physician–patient and interprofessional relationships
Apply core principles of medical ethics in clinical decision-making
Demonstrate ethical and professional conduct in rheumatology practice
Establish and maintain respectful, collaborative relationships with supervisors, multidisciplinary teams, and healthcare staff
Deliver accurate, appropriate, and patient-centered education to patients, families, and caregivers
<u>Second Year</u>
Incorporate legal and socioeconomic considerations into the assessment and management of rheumatic diseases
Make clinical decisions under adverse conditions with emotional regulation and effective leadership, while recognizing personal and professional limitations</td></tr>
<tr><td>Knowledge</td><td>Apply ethical principles in medical practice
Follow good clinical practice guidelines in rheumatology
Understand interprofessional teamwork and healthcare communication
Recognize sociocultural factors influencing rheumatologic care</td></tr>
<tr><td>Skills</td><td>Practice active listening and empathy
Communicate assertively with patients and healthcare teams
Participate effectively in interprofessional clinical discussions
Identify and manage ethical and communication conflicts</td></tr>
<tr><td>Attitudes</td><td>Respect patient dignity and autonomy
Maintain ethical, responsible, and respectful behavior
Demonstrate professionalism in all care settings
Show commitment to collaboration and lifelong learning</td></tr>
<tr><td>Contexts of practice</td><td>Outpatient and inpatient rheumatology care
Interprofessional team meetings
Complex clinical and ethical decision-making
Conflict situations involving patients, families, and teams</td></tr>
<tr><td>Risk level</td><td>Moderate risk. Although there is no direct physical harm, failures in ethics, communication, teamwork, and professionalism may negatively impact quality of care, patient trust, adherence, and relational safety.</td></tr>
<tr><td>Expected level of supervision</td><td>Level 5 – Supervise others</td></tr>
<tr><td>Developmental milestones</td><td><table>
<tr><th>Training Stage</th><th>Developmental Milestone</th><th>Expected Objectives and Competencies</th></tr>
<tr><td colspan="3"><u>First year</u></td></tr>
<tr><td>1st semester</td><td>Practice under direct supervision</td><td>Performs activities under direct supervision; identifies simple ethical dilemmas</td></tr>
<tr><td>2nd semester</td><td>Participation with occasional support</td><td>Performs with occasional support; demonstrates empathy and ethical conduct</td></tr>
<tr><td colspan="3"><u>Second year</u></td></tr>
<tr><td>1st semester</td><td>Practice under indirect supervision</td><td>Performs under indirect supervision; resolves minor conflicts and contributes to team decisions</td></tr>
<tr><td>End of residency</td><td>Independent and collaborative practice</td><td>Performs independently and collaboratively; demonstrates full professionalism and ethical leadership</td></tr>
</table></td></tr>
<tr><td>Assessment tools</td><td>Mini-CEX focused on attitudes
360-degree evaluation
OSCE with ethical or communication conflict simulations
Reflective narratives and portfolio
Interprofessional team assessment</td></tr>
<tr><td>Entrustment criteria</td><td>Demonstrates ethical behavior across different contexts</td></tr>
</table>

	• Communicates clearly, respectfully, and empathetically<br>• Recognizes and respects professional and personal boundaries<br>• Works collaboratively with patients and healthcare teams<br>• Makes decisions based on ethical principles and best practices<br>• Actively seeks, receives, and applies feedback

EPA Number	2
**EPA Title**	**Obtain a structured, comprehensive rheumatologic history**
**Brief Description**	The resident consistently elicits, synthesizes, and documents a structured and comprehensive rheumatologic history, accurately identifying articular and extra-articular manifestations, relevant risk factors, comorbidities, and prior treatments. The resident demonstrates patient-centered communication through empathy, active listening, and clear information gathering to support clinical reasoning and decision-making.
**Rationale**	Rheumatologic history-taking is essential for diagnostic hypothesis formulation and clinical decision-making. A systematic approach enhances patient safety, humanization of care, and diagnostic effectiveness.
**Competency domains**	• Patient Care<br>• Medical knowledge<br>• Practice-based learning and improvement<br>• Interpersonal and communication Skills<br>• Professionalism
**Competencies addressed (based on the Competency Matrix of the National Medical Residency Commission)**	*First year*<br>• Fundamentals of musculoskeletal anatomy and biomechanics<br>• History-taking and physical examination<br>• Diagnostic hypotheses<br>• Clinical epidemiology<br>• Proper medical documentation<br>• Medical ethics<br><br>*Second year*<br>• Patient education<br>• Epidemiology of rheumatic diseases<br>• Ethical clinical practice
**Knowledge**	• Principles of focused history-taking<br>• Clinical features of rheumatic diseases<br>• Articular and extra-articular semiology<br>• Diagnostic and classification criteria
**Skills**	• Conduct patient-centered interviews<br>• Identify articular and systemic symptoms<br>• Organize clinical information in medical records<br>• Use appropriate language.
**Attitudes**	• Empathy and active listening<br>• Ethical behavior<br>• Respect for diversity<br>• Professionalism.
**Contexts of practice**	• Outpatient clinics<br>• Inpatient care<br>• Clinical discussions
**Risk level**	Moderate risk: errors or omissions in history-taking may lead to diagnostic delay or inappropriate management.
**Expected level of supervision**	Level 5 – Supervises others
**Developmental milestones**	(see below)
**Assessment tools**	• Mini-CEX

Developmental milestones

Training Stage	Developmental Milestone	Expected Objectives and Competencies
First year		
1st semester	Reactive indirect supervision	Performs structured history-taking in common cases
2nd semester	Reactive indirect supervision	Performs a structured history in both common and complex cases
Second year		
1st semester	Remote supervision	Performs independently without direct supervision
End of residency	Supervision of others	Performs independently and provides guidance to other residents and medical students

	• Structured checklist<br>• Direct observation<br>• Reflective portfolio.
**Entrustment criteria**	• Clear and fluent history-taking<br>• Appropriate symptom identification<br>• Exploration of relevant past history<br>• Empathy and respect<br>• Complete clinical documentation.

**EPA number**	**3**
**EPA title**	**Perform and accurately interpret a systematic rheumatologic physical examination**
**Brief description**	The resident performs a comprehensive musculoskeletal examination*, accurately identifies clinically relevant findings, and integrates them to support diagnosis, disease monitoring, and management in rheumatology.
**Rationale**	The physical examination is a cornerstone of rheumatologic practice, allowing identification of key clinical signs such as synovitis, enthesitis, dactylitis, and structural changes. Mastery of this activity is essential for clinical reasoning, diagnostic definition, and therapeutic decision-making.
**Competency domains**	• Patient care<br>• Medical knowledge<br>• Practice-based learning and improvement<br>• Interpersonal and communication skills<br>• Professionalism
**Competencies addressed (based on the competency matrix of the national medical residency commission)**	*First year*<br>• Perform history-taking and physical examination in patients with musculoskeletal complaints<br>• Apply foundational knowledge of musculoskeletal anatomy and biomechanics<br>• Formulate differential diagnoses<br>• Maintain accurate medical records<br>• Apply ethical principles in patient care<br>*Second year*<br>• Establish respectful relationships with supervisors, healthcare teams, and staff<br>• Integrate ethical principles into rheumatologic practice
**Knowledge**	• Apply musculoskeletal anatomy and biomechanics<br>• Perform and interpret rheumatologic semiology<br>• Recognize and describe major clinical manifestations of rheumatic diseases
**Skills**	• Perform inspection, palpation, and specific examination maneuvers with appropriate technique<br>• Systematize the physical examination by body segments<br>• Document and communicate findings clearly in the medical record<br>• Correlate physical findings with diagnostic hypotheses
**Attitudes**	• Demonstrate empathy, respect, and active listening<br>• Act ethically and professionally<br>• Ensure patient safety during physical examination<br>• Protect patient privacy and dignity
**Contexts of practice**	• Outpatient clinics<br>• Inpatient wards<br>• Emergency settings<br>• Inpatient or outpatient consultations
**Risk level**	Moderate risk: errors in performance or interpretation of the physical examination may lead to diagnostic delay, unnecessary investigations, or inappropriate management. Requires ongoing supervision and structured feedback
**Expected level of supervision**	Level 5 – Supervises others
**Developmental milestones**	(see table below)

Developmental milestones

Training Stage	Developmental Milestone	Expected Objectives and Competencies
First year		
1st semester	Direct supervision	Observes preceptors; identifies anatomical structures; performs basic inspection, palpation, and range-of-motion assessment
2nd semester	indirect supervision	Performs complete musculoskeletal examination; applies specific maneuvers; interprets findings and integrates them into clinical reasoning.
Second year		
Throughout the year:	Independently; supervision of others	Supervises and teaches junior trainees; integrates physical examination findings into diagnostic and therapeutic planning.

**Assessment tools**	• Mini-CEX<br>• DOPS<br>• OSCE<br>• Simulation-based assessment<br>• Structured checklists<br>• Direct observation with feedback
**Entrustment criteria**	• Performs a complete and systematic physical examination safely and respectfully<br>• Accurately interprets findings in clinical context<br>• Formulates coherent diagnostic and management plans<br>• Demonstrates professionalism and effective communication

***Minimum core physical examination maneuvers for rheumatology residency, aligned with routine clinical practice and the most common differential diagnoses:**

**Spine**	**Hips / Pelvis / SIJ**	**Shoulders**	**Knees**
• Modified Schober test (lumbar flexion)<br>• Occiput-to-wall distance<br>• Tragus-to-wall distance<br>• Chest expansion measurement<br>• Cervical rotation (ROM)<br>• Straight Leg Raise (SLR) test (Lasègue)<br>• Spurling test<br>• Fingertip-to-floor distance	• Hip internal/external rotation (ROM)<br>• FABER test (Patrick)<br>• FADIR test<br>• FAIR test<br>• Trendelenburg test<br>• Gaenslen test<br>• Volkmann sign/test<br>• Thomas test	• Neer impingement test<br>• Hawkins–Kennedy test<br>• Jobe test (Empty Can)<br>• Patte test (Hornblower sign)<br>• Speed test<br>• Apprehension tests (anterior/posterior)<br>• Lift-off test (Gerber)	• Effusion assessment (bulge sign/ballottement)<br>• Patellar tap test<br>• Anterior drawer test<br>• Posterior drawer test<br>• Lachman test<br>• Varus stress test (LCL)<br>• Valgus stress test (MCL)<br>• McMurray test
**Elbows**	**Feet / Ankles**	**Hands / Wrists / Fingers**	**Other / General**
• Resisted wrist flexion test (medial epicondylalgia)<br>• Resisted wrist extension test (lateral epicondylalgia)<br>• Cozen test<br>• Mill test<br>• Tinel sign (cubital tunnel)<br>• Cubital tunnel compression test<br>• Elbow flexion test (60 sec)	• Forefoot squeeze test (metatarsal compression)<br>• Toe-walking test<br>• Foot inspection (arch, hallux valgus, deformities)<br>• Anterior drawer test (ankle)	• MCP squeeze test<br>• Phalen test<br>• Tinel sign (median nerve)<br>• Finkelstein test<br>• Tender & swollen joint count<br>• Wrist/finger extension strength (radial nerve)<br>• Froment sign (ulnar nerve)	• Thoracic outlet syndrome: Adson / costoclavicular / Wright<br>• Manual muscle strength testing (MMT)<br>• Peripheral pulse palpation<br>• Focused neuro exam: sensation / DTRs / gait<br>• TMJ assessment: opening / excursions / palpation / loading

**EPA number**	**4**
**EPA title**	**Manage outpatient care for patients with rheumatic diseases.**
**Brief description**	The resident must be able to provide comprehensive outpatient care for patients with rheumatic diseases, demonstrating clinical competence, sound judgment, and professionalism.
**Rationale**	Outpatient management is extremely important, given that most rheumatic conditions are chronic and require continuous follow-up
**Related competency domains**	• Patient care<br>• Medical knowledge<br>• Practice-based learning and improvement<br>• Interpersonal communication<br>• Professionalism<br>• Systems-based practice
**Competencies addressed (based on the Competency Matrix of the National Medical Residency Commission)**	<u>*First year*</u><br>• Elicit and integrate a focused history and physical examination in patients with musculoskeletal complaints, correlating clinical findings with rheumatic diseases<br>• Select, interpret, and synthesize ancillary investigations—particularly hematologic, immunologic, biochemical, and histopathological tests—for the diagnosis and longitudinal follow-up of rheumatic and bone diseases<br>• Formulate appropriate and prioritized differential diagnostic hypotheses based on clinical and investigative data<br>• Prescribe, monitor, and adjust pharmacological and non-pharmacological therapies for rheumatic diseases in accordance with current evidence and guidelines<br>• Demonstrate ethical, moral, social, and economic awareness in physician–patient interactions and in collaboration with the healthcare team<br>• Maintain clear, complete, and up-to-date medical records, documenting clinical findings, diagnostic reasoning, management plans, and disease evolution in compliance with professional and regulatory standards (CRM)

<table>
<tr><td></td><td>• Develop, communicate, and implement a therapeutic plan that is discussed with, understood by, and accepted by the patient and/or legal guardian
• Demonstrate understanding of the Brazilian Unified Health System (SUS), including its organizational structure, access pathways, and regulatory processes
• Appropriately request, coordinate, and integrate consultations with other specialists when clinically indicated
• Apply fundamental principles of medical ethics consistently in clinical practice

<u>Second year</u>
• Recognize, assess, and manage signs and symptoms at the interface with related specialties, including orthopedics, physical medicine and rehabilitation, pediatric rheumatology, radiology, clinical pathology, nephrology, and dermatology
• Demonstrate competence in the appropriate indication, prescription, and monitoring of biologic and targeted immunomodulatory therapies
• Provide and apply structured education to patients and caregivers, promoting disease understanding, treatment adherence, and shared decision-making
• Incorporate legal and socioeconomic considerations into clinical decision-making and the management of rheumatic diseases
• Diagnose and manage the most prevalent rheumatic diseases using evidence-based and guideline-supported approaches
• Deliver supportive care to patients and families, including the application of palliative care principles and end-of-life care when clinically appropriate
• Make sound clinical decisions under adverse conditions, maintaining emotional regulation and professionalism, demonstrating effective leadership, and recognizing personal and professional limitations</td></tr>
<tr><td>Knowledge</td><td>• Demonstrate a solid understanding of the epidemiology, clinical presentation, and therapeutic management of rheumatic diseases
• Apply specialized rheumatology expertise to enhance comprehensive, patient-centered care</td></tr>
<tr><td>Skills</td><td>• Perform and interpret rheumatologic history and physical examination, identifying relevant articular, extra-articular, and systemic manifestations
• Use instruments to assess disease activity and functional disability to monitor progression and therapeutic response
• Recognize alarm signs and potential complications of rheumatic diseases and their treatments, establishing appropriate management and timely referrals
• Develop, implement, and reassess individualized therapeutic plans based on scientific evidence and patient preferences
• Provide structured guidance to patients and families, promoting health education, treatment adherence, understanding of the disease, and the therapeutic plan
• Communicate clearly, empathetically, and ethically with patients, family members, caregivers, and members of the healthcare team
• Record in a complete, organized, and safe manner the history, physical examination, diagnostic hypotheses, management decisions, and clinical evolution in the electronic medical record, ensuring traceability and continuity of care
• Apply public health principles and implement strategies to improve the quality and safety of outpatient rheumatology care</td></tr>
<tr><td>Attitudes</td><td>• Work collaboratively to plan comprehensive patient care
• Demonstrate empathy toward the emotions of the team, patients, and caregivers</td></tr>
<tr><td>Situations/Contexts of Application</td><td>• Outpatient clinic</td></tr>
<tr><td>Associated risk</td><td>Moderate: Although outpatient care most often involves longitudinal and planned management, diagnostic errors, failures in monitoring, inappropriate medication use, or failure to recognize complications may negatively impact disease course and patient safety.</td></tr>
<tr><td>Expected Level of Supervision</td><td>Level 5 – Supervises others</td></tr>
<tr><td>Developmental milestones</td><td><table>
<tr><th>Training Stage</th><th>Developmental Milestone</th><th>Expected Objectives and Competencies</th></tr>
<tr><td colspan="3"><u>First year</u></td></tr>
<tr><td>1st semester</td><td>Reactive indirect supervision</td><td>Conducts focused history-taking and reviews complementary tests in the context of follow-up; participates in management discussions with the preceptor; understands the principles of longitudinal care and outpatient coordination</td></tr>
<tr><td>2nd semester</td><td>Reactive indirect supervision</td><td>Identifies exacerbations and comorbidities; initiates active discussion of therapeutic adjustments; participates in</td></tr>
</table></td></tr>
</table>

			coordinating multiprofessional care under guidance; learns to refer patients to other professionals when necessary
	Second year		
	1st semester	Remote supervision	Develops and implements individualized therapeutic plans based on evidence and patient preferences; adjusts management in new and follow-up cases, recognizing limits of practice; assesses therapeutic response safely; actively coordinates interdisciplinary care, discussing cases with specialists and preceptors when indicated.
	End of residency	Supervision of others	Leads outpatient case discussions in clinical meetings; manages longitudinal patient care comprehensively with full autonomy under global supervision; acts in care transitions, referring patients appropriately across different levels of the healthcare system.
**Suggested assessment tools**	• Mini-CEX, case discussions<br>• Simulation<br>• Portfolio<br>• 360° evaluation		
**Entrustment criteria**	• Critically discusses clinical cases, articulates rationale, and presents alternative approaches and solutions related to rheumatologic and interprofessional patient management.<br>• Places the interests of patients and populations at the center of healthcare delivery, with the aim of promoting health, disease prevention, and health equity across the lifespan.		

**EPA number**	**5**
**EPA title**	**Provide inpatient care for patients with rheumatic diseases, including ward care, emergency care, intensive care unit management, and consultative services to other specialties**
**Brief description**	The resident provides safe and effective hospital-based care for patients with rheumatic diseases across inpatient wards, emergency departments, and intensive care units, including the management of interdisciplinary consultations, while demonstrating sound clinical reasoning, progressive autonomy, and effective teamwork.
**Rationale**	Inpatient management of rheumatology patients involves acute conditions, clinical complications, treatment-related adverse events, and complex situations that require integration of knowledge and safe interprofessional practice.
**Related competency domains**	• Patient care<br>• Medical knowledge<br>• Practice-based learning and improvement<br>• Interpersonal communication<br>• Professionalism<br>• Systems-based practice
**Competencies addressed (based on the competency matrix of the national medical residency commission)**	*First year*<br>• Recognize and manage rheumatologic emergencies and urgent conditions<br>• Coordinate care from hospital admission through discharge, ensuring continuity of care through a structured discharge summary/report<br>• Appropriately request and integrate consultations with other specialties when indicated<br>• Develop and prescribe a therapeutic plan that is discussed with, understood by, and accepted by the patient and/or legal guardian, and maintain a clear, complete, and up-to-date medical record<br>• Apply relevant medicolegal principles in clinical practice<br>• Obtain informed consent when applicable, using clear and appropriate language<br>• Incorporate cost-awareness into clinical decision-making, considering cost–benefit when indicating tests and therapies, and demonstrate competence in pharmacological treatments used in rheumatology<br><br>*Second year*<br>• Assess and manage signs and symptoms at the interface with related specialties (e.g., orthopedics, radiology, nephrology, dermatology)<br>• Provide structured education and counseling for patients, family members, and caregivers<br>• Incorporate legal and socioeconomic considerations into clinical decision-making in rheumatic diseases<br>• Provide supportive care when appropriate, including principles of palliative and end-of-life care<br>• Make sound clinical decisions under adverse conditions, maintaining emotional control and professionalism, demonstrating leadership, and recognizing personal limits
**Knowledge**	• Apply and correlate musculoskeletal system anatomy and biomechanics

	• Perform and interpret osteoarticular semiology and that of other organs and systems<br>• Recognize and describe the main clinical manifestations of the organs and systems most involved in rheumatic diseases<br>• Interpret and use immunologic and autoimmunity markers involved in rheumatic diseases		
**Skills**	• Perform initial clinical assessment and severity stratification<br>• Formulate rapid and targeted diagnostic hypotheses<br>• Request appropriate tests in the hospital context<br>• Discuss cases with specialists and lead clinical decision-making<br>• Communicate with patients and families in higher-severity situations		
**Attitudes**	• Demonstrate ethical and safe conduct in the hospital environment<br>• Recognize personal limits and seek support when necessary<br>• Work collaboratively with the multidisciplinary team<br>• Maintain emotional balance in critical situations		
**Situations/contexts of application**	Inpatient wards, emergency departments, intensive care units, and consultative services in both inpatient and outpatient settings		
**Associated risk**	High: In the hospital setting, the resident frequently manages acute presentations, severe complications, high-risk medications, decompensated comorbidities, and time-sensitive clinical decisions. Errors may result in significant adverse outcomes, including life-threatening events		
**Expected level of supervision**	Level 5 – supervises others		
**Developmental milestones**	Training Stage	Developmental Milestone	Expected Objectives and Competencies
	First year		
	1st semester	Reactive indirect supervision	Conducts focused history-taking and reviews complementary tests in the context of follow-up; participates in management discussions with the preceptor; understands the principles of longitudinal care and outpatient coordination
	2nd semester	Reactive indirect supervision	Identifies exacerbations and comorbidities; initiates active discussion of therapeutic adjustments; participates in coordinating multiprofessional care under guidance; learns to refer patients to other professionals when necessary
	Second year		
	1st semester	Remote supervision	Develops and implements individualized therapeutic plans based on evidence and patient preferences; adjusts management in new and follow-up cases, recognizing limits of practice; assesses therapeutic response safely; actively coordinates interdisciplinary care, discussing cases with specialists and preceptors when indicated.
	End of residency	Supervision of others	Supervises and guides junior residents and students during outpatient follow-up; leads outpatient case discussions in clinical meetings; manages longitudinal patient care comprehensively with full autonomy under global supervision; acts in care transitions, referring patients appropriately across different levels of the healthcare system
**Suggested assessment tools**	• Mini-CEX, DOPS<br>• Inpatient case discussions<br>• Portfolio<br>• 360° evaluation.		
**Entrustment criteria**	• Makes safe and appropriate clinical decisions in inpatient care<br>• Discusses and justifies therapeutic alternatives based on clinical context and evidence<br>• Leads and coordinates interdisciplinary patient management effectively<br>• Appropriately recognizes situations that require specialized support or referral		

EPA number	6
**EPA title**	**Appropriately order and interpret diagnostic tests to support diagnosis and longitudinal monitoring of rheumatic diseases.**
**Brief description**	The resident judiciously orders complementary investigations based on the patient's clinical presentation and accurately interprets results to support diagnosis, guide longitudinal monitoring, and identify potential treatment-related adverse events. The resident communicates test results clearly, concisely, and in patient-appropriate language to the patient and, when applicable, to family members or the legal guardian.
**Rationale**	The appropriate indication and interpretation of laboratory test results, performed in conjunction with clinical assessment, are essential for clinical reasoning, diagnosis, and treatment planning. Ultimately, they improve healthcare quality and patient safety.
**Related competency domains**	• Patient care<br>• Medical knowledge<br>• Practice-based learning and improvement<br>• Professionalism<br>• Systems-based practice
**Competencies addressed (based on the competency matrix of the national medical residency commission)**	<u>*First year*</u><br>• Demonstrate competence in the indication and interpretation of ancillary tests—particularly hematologic, immunologic, biochemical, and histopathological investigations—for the diagnosis and follow-up of rheumatic and bone diseases<br>• Formulate appropriate differential diagnostic hypotheses.<br>• Maintain clear, complete, and legible medical records, documenting clinical data to support diagnostic reasoning and management decisions<br>• Apply fundamental principles of medical ethics in daily clinical practice<br>• Incorporate cost-awareness into clinical decision-making, using resources responsibly and in the patient's best interest while maintaining standards of excellence<br>• Apply cost–benefit principles when indicating medications and complementary tests, avoiding unnecessary or low-value investigations<br><br><u>*Second year*</u><br>• Appropriately integrate ancillary tests and therapeutic resources in the management of patients with complex clinical presentations<br>• Incorporate legal and socioeconomic considerations into the care of patients with rheumatic diseases<br>• Demonstrate competence in pharmacological therapies used in the treatment of rheumatic diseases
**Knowledge**	• Understand and apply the pathophysiology of rheumatic diseases<br>• Define and justify clinical indications for requesting complementary tests<br>• Interpret and analyze sensitivity, specificity, predictive values, false-positive/false-negative results, and interferences related to clinical conditions or medication use<br>• Evaluate and apply pharmacoeconomic concept
**Skills**	• Request tests appropriately, based on the clinical hypothesis<br>• Avoid ordering unnecessary tests that do not add relevant information to clinical reasoning<br>• Interpret test results according to the clinical hypothesis<br>• Screen for diseases and monitor disease activity based on test results<br>• Make clinical decisions: confirm, exclude, or revise diagnoses<br>• Monitor and propose therapeutic adjustments appropriately<br>• Communicate test results clearly, using simple language for the patient, family member, or legal guardian
**Attitudes**	• Order tests responsibly based on clear clinical justification, avoiding routine, automated, or low-value requests<br>• Demonstrate ethical and professional conduct by maintaining confidentiality and sharing information only with authorized members of the healthcare team<br>• Communicate laboratory results with respect and sensitivity, using clear and accessible language to ensure understanding by patients and, when appropriate, family members or legal guardians<br>• Collaborate respectfully with professionals involved in specimen collection, processing, and laboratory analysis<br>• Maintain up-to-date knowledge of emerging diagnostic methods and technologies relevant to rheumatology practice
**Situations/contexts of application**	• Outpatient clinic<br>• Inpatient ward<br>• Consultation/consultative services

<table>
<tr><td>Associated risk</td><td colspan="3">Moderate. Although this activity is limited to ordering and interpreting tests, errors at this stage may lead to misdiagnosis, delayed treatment, unnecessary interventions, or missed therapeutic opportunities, with potentially significant consequences for patient safety and clinical outcomes</td></tr>
<tr><td>Expected level of supervision</td><td colspan="3">Level 5 – supervises others</td></tr>
<tr><td rowspan="7">Developmental milestones</td><td>Training Stage</td><td>Developmental Milestone</td><td>Expected Objectives and Competencies</td></tr>
<tr><td colspan="3"><u>First year</u></td></tr>
<tr><td>1st semester</td><td>Indirectly supervised autonomy</td><td>Requests tests with simple clinical justification under supervision. Begins correlating laboratory abnormalities with diagnostic hypotheses. Identifies the most common laboratory abnormalities in rheumatic diseases. Recognizes the need for diagnostic complementation with preceptor support.</td></tr>
<tr><td>2nd semester</td><td>Indirectly supervised autonomy</td><td>Requests diagnostic tests requiring more complex clinical justification. Correlates laboratory abnormalities with diagnostic hypotheses and clinical context. Identifies less common and more complex laboratory abnormalities.</td></tr>
<tr><td colspan="3"><u>Second year</u></td></tr>
<tr><td>1st semester</td><td>Consolidation of supervised autonomy</td><td>Requests tests based on clinical hypotheses. Interprets results together with clinical manifestations. Recognizes limitations, interferences, and the possibility of false-positive/false-negative results. Discusses laboratory cases in clinical meetings. Identifies the need for specialized complementary tests.</td></tr>
<tr><td>End of residency</td><td>Supervision of others and leadership in longitudinal care</td><td>Guides junior residents in ordering and interpreting tests. Integrates laboratory findings into longitudinal patient assessment. Discusses cost–benefit of tests requested in different contexts. Participates in therapeutic decisions based on laboratory monitoring.</td></tr>
<tr><td>Suggested assessment tools</td><td colspan="3">• Mini-CEX
• Case discussion
• Structured checklist
• Direct observation with feedback
• Simulation</td></tr>
<tr><td>Entrustment criteria</td><td colspan="3">• Orders diagnostic tests based on clearly defined clinical questions and diagnostic hypotheses
• Avoids low-value or unnecessary testing, promoting the rational use of healthcare resources
• Integrates history and physical examination findings with laboratory data to support sound clinical reasoning
• Demonstrates accountability and progressive autonomy in the ordering and interpretation of diagnostic tests
• Interprets test results critically, considering sensitivity, specificity, predictive values, and test limitations
• Avoids making clinical decisions based solely on isolated laboratory abnormalities, ensuring appropriate clinical correlation
• Initiates, adjusts, discontinues, and monitors treatment based on accurate interpretation of results and longitudinal patient assessment
• Communicates test results clearly and confidently, using patient-appropriate language with patients and, when applicable, family members or legal guardians
• Demonstrates professionalism and effective collaboration within the multidisciplinary healthcare team</td></tr>
</table>

**EPA number**	**7**
**EPA title**	**Appropriately order and interpret imaging studies to confirm or refine diagnostic hypotheses in rheumatic diseases**
**Brief description**	The resident appropriately requests imaging studies and accurately interprets their findings based on the clinical hypothesis, integrating imaging results into diagnostic reasoning and the therapeutic plan.
**Rationale**	Appropriate indication and interpretation of imaging studies are essential in rheumatology practice for diagnosis, monitoring disease activity, and assessing structural damage. This activity contributes to diagnostic accuracy, rational use of resources, and improvement in quality of care.
**Related competency domains**	• Patient care<br>• Medical knowledge<br>• Practice-based learning and improvement<br>• Professionalism

<table>
<tr><td></td><td colspan="3">• Systems-based practice</td></tr>
<tr><td>Competencies addressed (based on the competency matrix of the national medical residency commission)</td><td colspan="3"><u>First year</u>
• Evaluate imaging techniques related to the musculoskeletal system, their indications, and interpretation: radiography, computed tomography, magnetic resonance imaging, ultrasonography, nuclear medicine, and others
• Develop differential diagnostic hypotheses
• Produce a legible medical record for each patient, containing clinical data to support appropriate case management
• Value the cost–benefit relationship for good practices in indicating medications and complementary tests

<u>Second year</u>
• Appropriately apply ancillary imaging tests and diagnostic resources in complex situations
• Discuss imaging studies with the multiprofessional team
• Value the legal and socioeconomic aspects of rheumatic diseases</td></tr>
<tr><td>Knowledge</td><td colspan="3">• Apply basic anatomy knowledge.
• Understand and apply the basic principles of imaging modalities (radiography, ultrasonography, magnetic resonance imaging, computed tomography, bone densitometry, scintigraphy, and others).
• Recognize and describe typical radiologic findings of rheumatic diseases.
• Indicate and analyze the limitations of each imaging modality.</td></tr>
<tr><td>Skills</td><td colspan="3">• Request imaging studies appropriate to the clinical hypothesis.
• Interpret reports and images in conjunction with clinical data.
• Discuss imaging studies with the multiprofessional team.
• Document findings clearly and in a structured manner.</td></tr>
<tr><td>Attitudes</td><td colspan="3">• Act responsibly when requesting imaging studies, avoiding unnecessary exams.
• Demonstrate respect for the patient when explaining the purpose of imaging studies.
• Maintain an ethical and collaborative attitude when discussing findings with the team.
• Seek continuous updating on imaging interpretation.</td></tr>
<tr><td>Situations/contexts of application</td><td colspan="3">• Outpatient clinic
• Inpatient ward
• Emergency department
• Intensive care unit
• Multidisciplinary clinical meetings</td></tr>
<tr><td>Associated risk</td><td colspan="3">Moderate risk: Errors in ordering or interpreting imaging studies may lead to incorrect diagnoses, delays in initiating appropriate treatment, or unnecessary procedures. In addition, inappropriate indication may expose the patient to unnecessary radiation or high-cost tests without clinical benefit.</td></tr>
<tr><td>Expected level of supervision</td><td colspan="3">Level 5 – supervises others</td></tr>
<tr><td rowspan="7">Developmental milestones</td><td>Training Stage</td><td>Developmental Milestone</td><td>Expected Objectives and Competencies</td></tr>
<tr><td><u>First year</u></td><td></td><td></td></tr>
<tr><td>1st semester</td><td>Indirectly supervised autonomy</td><td>Requests imaging studies with indirect supervision and justifies indications according to initial diagnostic hypotheses. Begins interpreting the most common findings under guidance. Identifies basic abnormalities on radiographs and ultrasonography.Recognizes limitations and requests preceptor support to discuss more complex imaging studies.</td></tr>
<tr><td>2nd semester</td><td>Indirectly supervised autonomy</td><td>Requests imaging studies with indirect supervision for more complex cases. Begins interpreting more complex findings.</td></tr>
<tr><td><u>Second year</u></td><td></td><td></td></tr>
<tr><td>1st semester</td><td>Consolidation of supervised autonomy</td><td>Requests and prioritizes imaging studies based on clinical course. Interprets findings on radiographs, ultrasonography, computed tomography, magnetic resonance imaging, densitometry, and scintigraphy with indirect supervision. Correlates imaging findings with disease activity, chronicity, and structural damage. Initiates imaging case discussions in clinical meetings.</td></tr>
<tr><td>End of residency</td><td>Supervision of others and leadership in longitudinal care</td><td>Supervises junior residents in ordering and interpreting imaging studies. Critically discusses indications and limitations of each imaging modality.</td></tr>
</table>

			Integrates imaging findings into longitudinal follow-up and therapeutic planning. Actively participates in complex clinical decisions based on imaging data.
**Suggested assessment tools**	• Mini-CEX<br>• Case discussion<br>• Structured checklist<br>• Direct observation with feedback<br>• Simulation<br>• OSCE		
**Entrustment criteria**	• Requests imaging studies appropriately based on diagnostic hypotheses<br>• Interprets imaging findings accurately and within the appropriate clinical context<br>• Discusses imaging results with the healthcare team, including the radiologist<br>• Explains imaging findings clearly to the patient<br>• Acts responsibly in the use of healthcare resources		

**EPA Number**	**8**
**EPA Title**	**Indicate and prescribe non-pharmacological treatment strategies in rheumatology**
**Brief Description**	The resident appropriately indicates, prescribes, guides, and monitors non-pharmacological interventions in the care of patients with rheumatic diseases—including physical therapy, occupational therapy, supervised physical activity, orthotic interventions, health education, lifestyle modifications, and psychosocial support—integrating these measures safely and individually into the therapeutic plan.
**Rationale**	Comprehensive management of rheumatic diseases goes beyond pharmacological therapy. Properly prescribed non-pharmacological interventions are essential for pain control, functional improvement, treatment adherence, quality of life, and reduction of disease impact. Competence in indicating, prescribing, and integrating these measures ensures patient-centered and multiprofessional care.
**Related Competency Domains**	• Medical knowledge<br>• Patient care<br>• Interpersonal communication<br>• Professionalism<br>• Evidence-based practice<br>• Teamwork
**Competencies addressed (based on the Competency Matrix of the National Medical Residency Commission)**	*First year*<br>• Demonstrate competence in providing structured education and counseling to patients, family members, and caregivers<br>• Value and request consultations with other specialists<br>• Analyze the costs of medical practice and use them for the patient's benefit, maintaining standards of excellence<br><br>*Second year*<br>• Assess and manage signs and symptoms that interface with related specialties, with emphasis on Physical Medicine and Rehabilitation<br>• Demonstrate competence in providing structured education and counseling to patients, family members, and caregivers<br>• Provide supportive care for patients and families in palliative medicine and end-of-life care
**Knowledge**	• Fundamentals of rehabilitation in rheumatology.<br>• Principles of physical therapy, occupational therapy, and physical activity.<br>• Impact of nutrition, sleep, mental health, and social support on rheumatic diseases.<br>• Scientific evidence supporting non-pharmacological interventions.
**Skills**	• Indicate and prescribe appropriate physical activity and therapeutic exercises.<br>• Refer to and prescribe physical therapy, occupational therapy, psychology, nutrition, and speech therapy when indicated.<br>• Assess, prescribe, and recommend supportive devices (orthoses, functional adaptations).<br>• Conduct and prescribe health education focused on self-care<br>• Integrate non-pharmacological prescriptions into the multiprofessional therapeutic plan.
**Attitudes**	• Encourage adherence to prescribed non-pharmacological measures.<br>• Promote patient autonomy in self-care.<br>• Value multiprofessional collaboration.<br>• Respect cultural and individual preferences.

**Situations/Contexts of Application**	Outpatient clinics; inpatient wards; multiprofessional rehabilitation; health education groups; interdisciplinary meetings.		
**Associated Risk**	Moderate risk: although generally safe, poorly indicated or prescribed non-pharmacological measures (e.g., physical activity without adequate supervision, inappropriate use of orthoses, failures in multiprofessional integration) may cause physical overload, functional deterioration, or delays in case management.		
**Expected Level of Supervision**	Level 5 – Supervises others		
**Developmental milestones**	Training Stage	Developmental Milestone	Expected Objectives and Competencies
	First year		
	1st semester	Indirect supervision	Recognizes non-pharmacological treatment options and learns to prescribe them with preceptor support
	2nd semester	Indirect supervision	Indicates and prescribes simple measures (educational guidance, basic referrals).
	Second year		
	1st semester	Indirect supervision	Prescribes and integrates non-pharmacological measures into the therapeutic plan in complex cases
	End of residency	Supervision of others and full autonomy	Demonstrates autonomy to prescribe, monitor, and guide residents in non-pharmacological strategies.
**Suggested Assessment Tools**	• Mini-CEX<br>• DOPS (for referrals/orthoses)<br>• Reflective portfolio<br>• Multiprofessional team case discussion<br>• Structured feedback		
**Criteria to Consider as "Entrustable"**	• Up-to-date knowledge of non-pharmacological therapies<br>• Appropriate and individualized indication and prescription<br>• Clear communication with the patient and the team<br>• Complete and objective clinical documentation<br>• Integration of multiprofessional care		

**EPA Number**	**9**
**EPA Title**	**Indicate, prescribe, and monitor pharmacological treatment in rheumatology**
**Brief Description**	The resident appropriately indicates, prescribes safely, and monitors the therapeutic efficacy and adverse effects of medications used in rheumatology, considering risks, benefits, cost-effectiveness, and individual patient characteristics.
**Rationale**	Pharmacological therapy is a fundamental pillar in the care of rheumatology patients. Rational, safe, and monitored use of medications is essential to ensure therapeutic efficacy, reduce risks, prevent complications, and promote adherence, always in accordance with the best scientific evidence and clinical guidelines.
**Related Competency Domains**	• Medical knowledge<br>• Patient care<br>• Patient safety<br>• Evidence-based practice<br>• Systems-based practice<br>• Clinical decision-making<br>• Interpersonal communication<br>• Professionalism<br>• Continuous learning and improvement
**Competencies addressed (based on the Competency Matrix of the National Medical Residency Commission)**	*First year*<br>• Apply evidence-based pharmacological therapies for the treatment of rheumatic diseases, ensuring safe prescribing and appropriate monitoring<br>• Prescribe the therapeutic plan, informed and accepted by the patient and/or legal guardian<br>• Value the cost–benefit relationship for good practices in indicating medications and complementary tests<br><br>*Second year*<br>• Demonstrate competence in the indication, prescription, and monitoring of biologic and targeted immunomodulatory therapies.<br>• Demonstrate competence in the diagnosis and management of the major rheumatic diseases (e.g., rheumatoid arthritis, spondyloarthritis, systemic lupus erythematosus, vasculitis), with appropriate therapeutic selection and longitudinal follow-up.<br>• Value the legal and socioeconomic aspects of rheumatologic diseases.

**Knowledge**	• Know the indications, doses, regimens, and routes of administration of rheumatologic medications<br>• Explain mechanisms of action and drug interactions<br>• Analyze clinical and laboratory parameters for monitoring efficacy and safety<br>• Identify early adverse events and infectious, metabolic, and hematologic complications<br>• Describe legal and institutional regulations related to the use of special medications<br>• Describe mechanisms for technology incorporation in SUS and in supplementary health systems
**Skills**	• Indicate and prescribe medications in an individualized, safe, and ethical manner according to national guidelines<br>• Recognize and indicate vaccination for patients in special situations (e.g., immunocompromised patients, older adults)<br>• Recognize major adverse events and manage them appropriately<br>• Adjust dosing according to clinical response, comorbidities, and adverse events<br>• Request and interpret monitoring tests<br>• Counsel patients regarding correct medication use, risks, and benefits<br>• Document pharmacological management clearly and completely in the medical record
**Attitudes**	• Take responsibility for the use of potentially toxic medications<br>• Demonstrate empathy and clarity when communicating risks, benefits, and therapeutic alternatives<br>• Maintain continuous vigilance in the follow-up of patients receiving pharmacological therapies<br>• Commit to continuous scientific updating<br>• Respect ethical, legal, and institutional standards
**Situations/Contexts of Application**	Inpatient wards, outpatient clinics, infusion centers, clinical pharmacy, intensive care units, and multiprofessional discussions.
**Associated Risk**	High risk: errors in indication, prescribing, or monitoring may result in serious complications (infections, metabolic, hematologic, or organ toxicities), with potential irreversibility or risk of death.
**Expected Level of Supervision**	Level 5 – Supervises others

Developmental milestones

Training Stage	Developmental Milestone	Expected Objectives and Competencies
First year		
1st semester	Indirectly supervised prescribing	Participates in therapeutic choice with preceptor guidance; prescribes under indirect review; applies initial safety monitoring
2nd semester	Indirectly supervised prescribing	Prescribes in common situations with reactive supervision; identifies and reports early adverse events; demonstrates responsibility in medication use
Second year		
1st semester	Indirectly supervised prescribing	Indicates and prescribes individualized medications according to disease severity and comorbidities; requests and interprets monitoring tests; discusses complex management under indirect supervision
End of residency	Supervision of others and full autonomy	Supervises junior residents; evaluates and manages complex adverse effects; proposes dose adjustments or therapeutic switches; works with the multiprofessional team and participates in clinical audits and institutional decisions

**Suggested Assessment Tools**	• Mini-CEX<br>• Case discussions<br>• Prescribing checklist<br>• Portfolio<br>• 360° evaluation.
**Criteria to Consider as “Entrustable”**	• Prescribes immunosuppressive therapies safely and appropriately.<br>• Correctly monitors toxicity and adverse events.<br>• Counsels patients regarding medication use and potential adverse events.<br>• Acts with ethical responsibility in therapeutic indications.

**EPA Number**	**10**
**EPA Title**	**Perform arthrocentesis and joint and periarticular injections**
**Brief Description**	The resident competently performs arthrocentesis and intra-articular injection procedures in patients with rheumatic diseases
**Rationale**	Arthrocentesis is a minimally invasive procedure used to evaluate synovial fluid, a constituent of synovial joints, providing information about inflammatory and/or infectious processes affecting the joints.

<table>
<tr><td></td><td colspan="3">Joint injection is a medical procedure whose main objectives are pain relief, reduction of inflammation, and improvement of joint function, reducing or avoiding the use of systemic medications.</td></tr>
<tr><td>Related Competency Domains</td><td colspan="3">• Patient care
• Medical knowledge
• Practice-based learning and improvement
• Interpersonal communication
• Professionalism</td></tr>
<tr><td>Competencies addressed (based on the Competency Matrix of the National Medical Residency Commission)</td><td colspan="3"><u>First year</u>
• Demonstrate a solid understanding of the anatomy, biochemistry, physiology, biomechanics, and histology of the musculoskeletal system
• Elicit and perform a focused history and physical examination in patients with musculoskeletal complaints, including comprehensive semiologic assessment of rheumatic manifestations
• Demonstrate competence in performing arthrocentesis and joint and periarticular injection procedures
• Analyze and interpret synovial fluid findings to support diagnostic reasoning and clinical decision-making.
• Obtain informed consent from the patient or legal representative through clear, appropriate, and comprehensible communication prior to procedures
<u>Second year</u>
• Perform procedures autonomously in more complex joints
• Recognize early signs of post-procedure complications
• Supervise junior colleagues in simple procedures</td></tr>
<tr><td>Knowledge</td><td colspan="3">• Apply knowledge of musculoskeletal anatomy and biomechanics
• Know the indications for arthrocentesis and joint/periarticular injections
• Understand the results of synovial fluid analysis
• Understand and apply the pharmacology of intra-articular medications</td></tr>
<tr><td>Skills</td><td colspan="3">• Prepare the necessary materials for arthrocentesis and injections (needles, syringes, antiseptics, anesthetics, corticosteroids, sterile materials)
• Perform correct antisepsis and field preparation
• Execute puncture and/or injection techniques in peripheral joints and bursae
• Recognize and aspirate synovial fluid adequately for analysis
• Handle and store synovial fluid samples correctly for laboratory submission
• Select and perform the appropriate type of injection (intra-articular, periarticular, intralesional)
• Document the procedure clearly, including justification, technique used, medication, dose, immediate response, and complications</td></tr>
<tr><td>Attitudes</td><td colspan="3">• Demonstrate empathy toward the patient
• Ensure patient comfort during the procedure is a priority
• Perform the procedure according to patient safety standards
• Select appropriate materials and medications
• Keep the patient under observation after the procedure</td></tr>
<tr><td>Situations/Contexts of Application</td><td colspan="3">Outpatient clinic, inpatient ward, emergency department, intensive care unit</td></tr>
<tr><td>Associated Risk</td><td colspan="3">High risk: Although relatively frequent in rheumatology practice, it involves direct patient risks such as iatrogenic joint infection, bleeding, neurovascular injury, severe pain, adverse reactions to intra-articular medications, and technical failures. Safe performance requires detailed anatomical knowledge, strict aseptic technique, progressive manual skill acquisition, and qualified supervision, especially in the early phases of training.</td></tr>
<tr><td>Expected Level of Supervision</td><td colspan="3">Level 5 – Supervises others</td></tr>
<tr><td rowspan="4">Developmental Milestones (Milestones</td><td>Training Stage</td><td>Developmental Milestone</td><td>Expected Objectives and Competencies</td></tr>
<tr><td><u>First year</u></td><td></td><td></td></tr>
<tr><td>1st semester</td><td>Indirectly supervised prescribing
(In some scenarios, depending on preceptor evaluation, may begin directly supervised execution).</td><td>Observes procedures performed by preceptors. Understands indications, contraindications, materials, and techniques for arthrocentesis and injections. Learns basic principles of asepsis and local analgesia. Studies joint anatomy applied to procedures.</td></tr>
<tr><td>2nd semester</td><td>Start of directly supervised execution</td><td>Performs joint aspirations in large joints (knee, shoulder) under direct supervision. Performs simple injections with in-person guidance. Correctly identify anatomical planes. Learns to recognize technical difficulties and when to stop the procedure.</td></tr>
</table>

	Second year		
	1st semester	Execution with progressively indirect supervision	Performs arthrocentesis and injections in various joints (including wrist, ankle, and small joints) with indirect supervision.<br>Manages mild complications during the procedure. Selects cases appropriately, considering risk–benefit and contraindications. Documents the procedure with quality and safety.
	End of residency	Supervises others and acts with technical autonomy	Supervises junior colleagues in simple procedures. Performs autonomously procedures in more complex joints (posterior shoulder, image-guided hip, if available).<br>Recognizes early signs of post-procedure complications. Acts safely and confidently when performing indicated procedures.
**Suggested Assessment Tools**	• DOPS (Direct Observation of Procedural Skills)<br>• Logbook (procedure count tracking)		
**Criteria to Consider as "Entrustable"**	• Correctly identifies the need for arthrocentesis and/or joint injection<br>• Performs an adequate physical examination including correct procedure site identification (ultrasound-guided or not)<br>• Adequate patient preparation.<br>• Adequate material preparation<br>• Completes the procedure fully<br>• Collect material when indicated<br>• Requests and interprets synovial fluid tests<br>• Provides post-procedure patient guidance<br>• Follows up to identify procedure-related complications		

**EPA Number**	**11**
**EPA Title**	**Promote patient safety and quality of care, and provide patient and family education on rheumatic disease and its treatment**
**Brief Description**	The resident develops and implements risk management strategies focused on patient safety, aiming to prevent incidents that may result in harm.<br>The resident educates patients and their families about rheumatic diseases, potential complications, and evidence-based treatment options.
**Rationale**	Alignment of medical training with international goals and the National Patient Safety Program. Improvement of healthcare quality, promotion of autonomy and active participation of patients and families in therapeutic planning, and safety in care delivery.
**Related Competency Domains**	• Patient care<br>• Practice-based learning and improvement<br>• Interpersonal communication<br>• Professionalism<br>• Systems-based practice
**Competencies addressed (based on the Competency Matrix of the National Medical Residency Commission)**	*First year*<br>• Develop and implement a therapeutic plan that is discussed with, understood by, and accepted by the patient and/or legal guardian<br>• Apply fundamental principles of medical ethics in clinical practice<br>• Incorporate relevant medicolegal principles into professional practice<br>• Use healthcare resources responsibly, considering costs and applying them in the patient's best interest while maintaining standards of excellence<br>• Apply cost–benefit principles when indicating medications and complementary tests, promoting high-value care<br>• Provide structured education and counseling to patients, family members, and caregivers<br>• Maintain clear, accurate, and up-to-date medical records that appropriately document clinical care<br>• Obtain informed consent through clear and appropriate communication with the patient or legal representative prior to interventions or treatments<br><br>*Second year*

<table>
<tr><td></td><td>Incorporate legal and socioeconomic considerations into the management of rheumatic diseases.
Provide supportive care for patients and families, including principles of palliative and end-of-life care when appropriate
Demonstrate respect for the ethical, moral, social, and economic principles that guide the physician–patient relationship and collaboration with other healthcare professionals
Demonstrate understanding of the Brazilian Unified Health System (SUS), including its structure, organization, and regulatory framework
Facilitate shared decision-making, actively engaging patients and family members in the therapeutic planning process</td></tr>
<tr><td>Knowledge</td><td>Know and apply the National Patient Safety Program
Apply principles of medical ethics in daily clinical practice
Interpret and use concepts of clinical epidemiology in patient care
Analyze and apply pharmacoeconomics concepts in therapeutic decision-making
Conduct shared decision-making with the patient and family members</td></tr>
<tr><td>Skills</td><td>Communication skills
Provide patient education using objective and clear language, facilitating understanding of the disease and treatment measures
Validate patient questions and feelings, encouraging active participation in care
Provide information about diagnosis, disease course, and prognosis in an empathic manner
Use technology for information and communication
Document patient and family education clearly and completely
Use safety protocols and institutional pathways for referral and follow-up
Ensure confidentiality and respect for patient privacy at all stages</td></tr>
<tr><td>Attitudes</td><td>Understand and share the feelings of the patient and family members
Demonstrate a positive outlook regarding the disease under follow-up
Demonstrate dedication and responsibility in the care provided
Value patient progress at each stage of treatment
Respect the decisions of the patient and family members
Responsible and evidence-based use of artificial intelligence in patient care</td></tr>
<tr><td>Situations/Contexts of Application</td><td>Outpatient clinic, inpatient ward, intensive care unit, emergency department</td></tr>
<tr><td>Associated Risk</td><td>Moderate risk: the activity has an important impact on quality of care and may generate patient risk, but without imminent risk of death or irreversible harm.</td></tr>
<tr><td>Expected Level of Supervision</td><td>Level 5 – Supervises others</td></tr>
<tr><td>Developmental Milestones</td><td><table>
<tr><th>Training Stage</th><th>Developmental Milestone</th><th>Expected Objectives and Competencies</th></tr>
<tr><td>First year</td><td></td><td></td></tr>
<tr><td>1st semester</td><td>Introduction to safety concepts and initial communication</td><td>Understands basic principles of patient safety and quality of care. Provides education to patients and families. Participates in discussions on risks, prevention of adverse events, and communication of bad news. Learns basic strategies for empathic communication and accessible language.</td></tr>
<tr><td>2nd semester</td><td>Active participation in education under direct supervision</td><td>Provides patient and family education under direct supervision. Applies patient-centered communication strategies. Identifies risk factors for adverse events and discusses prevention strategies with the team. Participates in meetings discussing critical events or near misses</td></tr>
<tr><td>Second year</td><td></td><td></td></tr>
<tr><td>1st semester</td><td>Practice with indirect supervision and focus on safe care</td><td>Provides education with progressive autonomy, adapting language to the sociocultural profile of patients. Identifies potential care risks early and proposes improvement measures. Applies safe practices in prescribing, administering therapies, and transitions of care.</td></tr>
<tr><td>End of residency</td><td>Leadership in patient safety and therapeutic education</td><td>Supervises junior residents in patient education and identification of care risks. Participates in audits and institutional safety or quality committees. Develops and discusses proposals for continuous improvement of care processes. Acts</td></tr>
</table></td></tr>
</table>

<table>
<tr><td></td><td></td><td></td><td>as a facilitator in building a shared therapeutic plan with patients and families.</td></tr>
<tr><td>Suggested Assessment Tools</td><td colspan="3">Mini-CEX
Direct observation with feedback
Reflective portfolio</td></tr>
<tr><td>Criteria to Consider as “Entrustable”</td><td colspan="3">Identifies patient safety failures and acts to improve processes
Provides education and identifies adverse events resulting from treatment
Engages in risk management
Provides care mediated by technologies to expand access in the local context
Organizes their schedule to meet demands
Demonstrates respect, compassion, and empathy
Conveys confidence
Demonstrates professionalism with the multidisciplinary team
Works interprofessionally with the multidisciplinary team
Manages conflicts in the interprofessional practice environment
Meets patient comfort needs
Rationally explains proposed tests and treatments
Develops person-centered care plans (shared decision-making)
Conducts community-based health education</td></tr>
</table>
